# Poster Session II - A291 COMPARATIVE EFFICACY AND SAFETY OF IL-23P19 INHIBITORS IN INFLAMMATORY BOWEL DISEASE: A SYSTEMATIC REVIEW AND NETWORK META-ANALYSIS OF RANDOMIZED CONTROL TRIALS

**DOI:** 10.1093/jcag/gwaf042.291

**Published:** 2026-02-13

**Authors:** B Nguyen, S Quon, E Hazan, S Singh

**Affiliations:** The University of British Columbia Department of Medicine, Vancouver, BC, Canada; The University of British Columbia Department of Medicine, Vancouver, BC, Canada; The University of British Columbia Division of Gastroenterology, Vancouver, BC, Canada; The University of British Columbia Division of Gastroenterology, Vancouver, BC, Canada

## Abstract

**Background:**

Interleukin-23 (IL-23)p19 inhibitors are an important modality for management of moderate-severe inflammatory bowel disease (IBD), yet intra-class comparative data remains limited with no direct head-to-head trials comparing IL-23p19 inhibitors within class. We conducted a network meta-analysis (NMA) comparing the efficacy and safety of guselkumab, mirikizumab, and risankizumab in Crohn’s disease (CD) and ulcerative colitis (UC).

**Aims:**

To compare induction and maintenance outcomes in IBD across IL-23p19 inhibitors.

**Methods:**

Following PRISMA-NMA guidelines, 5 databases were searched to July 2025 for randomized control trials. Outcomes included clinical response/remission, endoscopic response/remission, and adverse events (AEs). Random-effects NMAs were used to estimate relative risks (RRs) with 95% confidence intervals (CIs). Non-randomized studies were excluded.

**Results:**

Fifteen trials (n = 11,166) formed connected networks including IL-23p19 agents, placebo, and ustekinumab. Across IBD subtypes, all IL-23p19 inhibitors were superior to placebo for clinical and endoscopic outcomes. In CD, guselkumab ranked highest in clinical response among biologic-exposed patients (RR 2.89, 95% CI 1.60-5.21) and achieved the largest effect in endoscopic remission (RR 6.57, 4.04-10.70). However, among biologic-naïve patients, ustekinumab led in clinical response (RR 2.37, 1.44-3.90). In UC, guselkumab showed the greatest efficacy for clinical remission (RR 2.67, 1.92-3.73), and mirikizumab ranked highest for endoscopic response (RR 3.47, 1.45-8.28). Overall AE rates were similar across agents, though serious AEs were lower with mirikizumab (RR 0.56, 0.40-0.77) and guselkumab (RR 0.62, 0.46-0.84).

**Conclusions:**

IL-23p19 inhibitors are effective for both induction and maintenance therapy in IBD. Within this class of biologics, Guselkumab demonstrates the most consistent and robust efficacy across outcomes, while mirikizumab offers a favourable safety profile and the strongest endoscopic healing in UC.

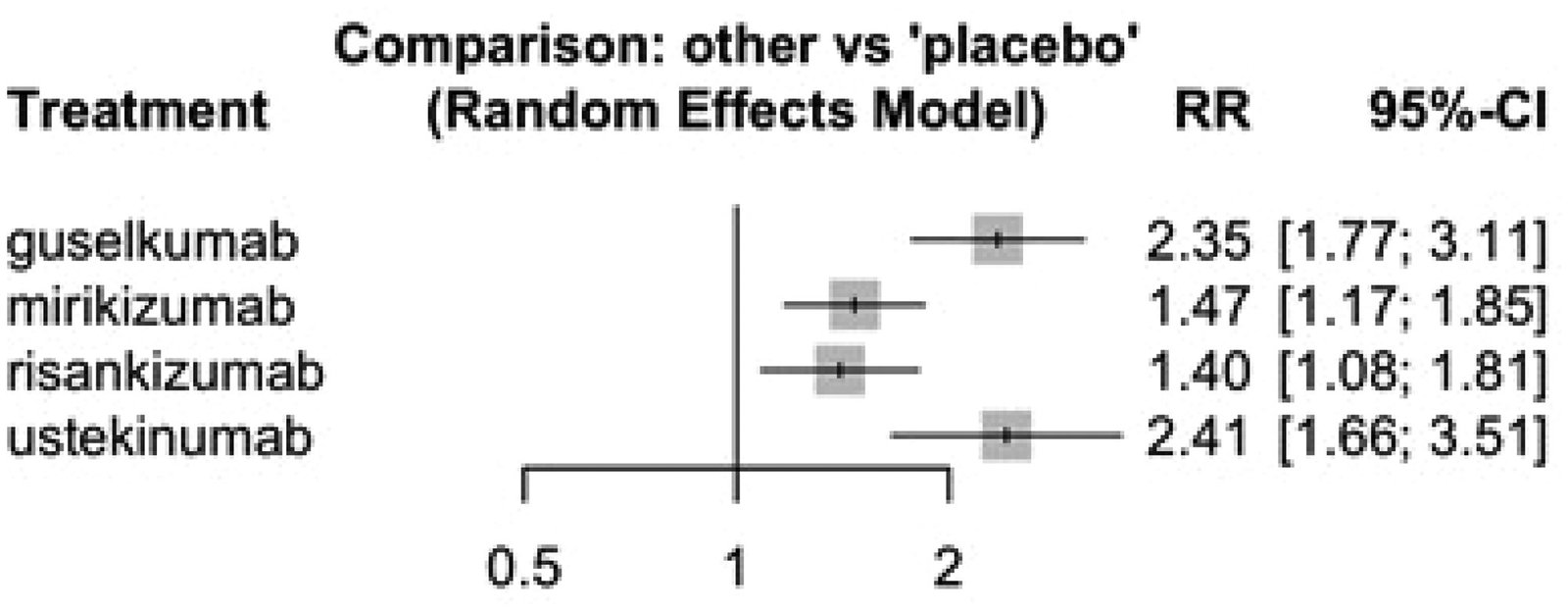

Relative risks (RR) for clinical response to IL-23p19 inhibitors compared to placebo in Crohn’s disease. In head-to-head comparisons, guselkumab was significantly more effective than mirikizumab (1.60 [1.11-2.29]) and risankizumab (1.67 [1.14-2.45]), but comparable to ustekinumab (0.97 [0.73-1.29]).

**Funding Agencies:**

None

